# Iranian nurses' perception of the public image of nursing and its association with their quality of working life

**DOI:** 10.1002/nop2.892

**Published:** 2021-05-05

**Authors:** Fariborz Roshangar, Amin Soheil, Golshan Moghbeli, Taneal Wiseman, Hossein Feizollahzadeh, Neda Gilani

**Affiliations:** ^1^ Department of Medical‐Surgical Nursing School of Nursing and Midwifery Tabriz University of Medical Sciences Tabriz Iran; ^2^ Department of Nursing Khoy University of Medical Sciences Khoy Iran; ^3^ Department of Medical‐Surgical Nursing School of Nursing and Midwifery Student Research Committee Tabriz University of Medical Sciences Tabriz Iran; ^4^ Susan Wakil School of Nursing and Midwifery Sydney Nursing School Melborn Vic. Australia; ^5^ Department of Statistics and Epidemiology School of Health Tabriz University of Medical Sciences Tabriz Iran

**Keywords:** Iran, nurses, nursing, public image of nursing, quality of nursing work life

## Abstract

**Aim:**

To investigate the association between the nurses’ perception of the public image (PI) of nursing and the quality of nursing work life (QNWL).

**Design:**

A descriptive correlational study.

**Methods:**

250 nurses of 12 hospitals affiliated with Tabriz University of Medical Sciences were sampled using a proportionate stratified sampling technique. Porter Nursing Image Scale and Brooks QNWL Scale were used for collecting data.

**Results:**

There was a significant positive correlation between nurses’ perception of their public image and QNWL (*r* = .158, *p *= .012). Nurses’ perception of their PI along with other significant predictors including gender, age, position, work shifts, residency, financial status, level of family support, spouse's education and spouse's job significantly explained 15.2% of the predictability of QNWL (*F*_(10,175)_ = 3.017, *p *= .001). Findings imply that enhancement of nurses' psychological status (nurses' perception of the public image of their profession) may improve their functional status (quality of nursing work life).

## 1 INTRODUCTION

The public image (PI) of nursing, which incorporates beliefs, ideas and impressions that people have of nurses and nursing, has always been a socio‐cultural issue for this profession (Emeghebo, 2012, Wallace, 2007). Recently, the environment in which nurses do practice has changed and this change foreshadows significant evolutions in the nursing profession (Maxwell, 2015). There is ample evidence that the PI of nursing has improved globally, especially in developed countries, which holds high value and reputation for the nursing profession. However, there still remains some negative view with regard to the PI of nurses (Meadus & Twomey, 2007; ten‐Hoeve et al., 2014). For example, in a recent integrative review, Glerean et al., (2017) found that young people's perceptions of the nursing profession were outdated, partly unrealistic and did not reflect the tasks of a modern nurse. According to the results of this review, these perceptions were largely influenced by social factors such as family and relatives, friends, media and significant others (Glerean et al., 2017). In developing countries, the public has a stereotypical view of nursing, in which nurses may be regarded as less intelligent than doctors, dependent on doctors, powerless and underpaid (Azadi et al., 2013; Price et al., 2013). This view, however, is not an accurate portrayal and is fuelled by misconception, misinformation and in some instances, by the actual realities of the profession (Cowin & Hengstberger‐Sims, 2006; Jinks & Bradley, 2004).

In Iran, nurses are striving to gain respect from the public; however, they are fighting to develop from a subordinate position inherited over the years (Farsi et al., 2010; Nasrabadi et al., 2004, Salsali, 2000). Many Iranian nurses suffered from a poor PI and a low social status which resulting in feelings of frustration, general dissatisfaction, hopelessness and confusion about self‐image and social identity (Nasrabadi et al., 2003). According to Varaei et al. (2012), in Iranian health care, the public often holds a negative stereotypical image of nursing, nurses and the role they play. This negative stereotypical image contributes to nurses’ perception that their work is not appreciated or respected (Varaei et al., 2012). Similarly, Vaismoradi et al. (2011) identified the lack of a clear and acceptable PI of nursing as one of the main barriers to the development of nurses' professional identity (Vaismoradi et al., 2011). In another qualitative study, Valizadeh et al. (2014) discovered that the public considers nursing as a feminine, physician‐subordinate and unrecognized career in Iran. They also identified that the feminine image of a “nurse” is a potential barrier for men considering nursing as a career and the majority of male nurses experienced significant personal, family and community resistance to entering a nursing profession (Valizadeh et al., 2014).

Overall, literature suggests that the PI of nursing impacts nurses' psychological and functional states. A positive PI of nursing is one factor which reflects high‐quality nursing care and is recognized by nurses and other groups for the difference it makes to patients' well‐being and will contribute to the empowerment of the profession (Takase et al., 2002, Ulmer, 2000). On the other hand, a poor PI of nursing is one of the biggest barriers to professionalization (Habibzadeh et al., 2013), professional socialization (Valizadeh et al., 2016b), professional autonomy (Roshanzadeh et al., 2018) and professional identity (Valizadeh & Ghorbani, 2016) of nursing in Iran. A poor PI of nursing may negatively impact not only the recruitment and retention, but also the attitudes towards work, work behaviours and performance of those in the nursing profession (Takase et al., 2002,2006, Wallace, 2007). Likewise, a number of studies have contended that the social world nurses live in has an impact on nurses' professional world. They have reported that the quality of nursing work life (QNWL) is seriously influenced by the PI of the nursing profession (Mohammadi et al., 2011; Takase et al., 2002; Zamanzadeh et al., 2013). However, this assumption has not yet been explored sufficiently and the extent of this association is still uncertain due to a lack of evidence.

According to the O’Brien‐Pallas and Baumann framework used by Brooks, QNWL is the degree to which nurses are able to satisfy important personal needs through their experiences in their work organization while achieving the organization's goals. The framework is synthesized into four dimensions: (a) work life‐home life, (b) work design, (c) work context and (d) work world (Brooks & Anderson, 2005). Based on the socio‐technical systems theory, assessing QNWL focuses on identifying opportunities for nurses to improve their work and work environment and affords organizations an opportunity to understand how work environments, work design, societal influences, and work and home life balance issues impact nurses’ working life and ultimately organizational productivity. Hence, assessing QNWL reveals areas for improvement where the needs of both the employees and the organization converge (Brooks et al., 2007).

Therefore, given the importance of these two issues and their direct or indirect effects on the quality of health care, it is imperative to investigate different dimensions of nurses’ perception of their PI and the extent to which this perception is associated with QNWL in Iran. Since the status of the nursing profession, nurses' perceptions, PI of nursing profession and QNWL may differ among cultures and countries, it is anticipated that the findings of this study will improve our knowledge and understanding of similar contexts with evolving healthcare systems that differ from the United States and the European systems. This study aimed to examine the nurses' perception of the PI of nursing and its association with QNWL in the Iranian context.

### 1.1 The healthcare system and nursing in Iran

Iran is a developing country located in the Middle East of Asia with a population of approximately 83 million in 2020 (Sadeghi et al., 2020). The Iranian healthcare system is based on three pillars: the public‐governmental system, the private sector and non‐governmental organizations. It is highly centralized and almost all of the decisions about general goals, policies and allocation of resources for an extensive healthcare network that offers basic healthcare services in addition to public health programmes, such as primary, secondary and tertiary health care, and medical education are made at the national level by the Ministry of Health and Medical Education (MOHME) in the Iranian government (Azizi, 2007). The ministry consists of nine deputies as follows: deputy for food and drugs; deputy for research and technology; deputy for health; deputy for education; deputy for culture and students’ affairs; deputy for treatment; deputy for legal, parliamentary and provincial affairs; deputy for management development, resources and planning; and deputy for nursing (Mosadeghrad & Rahimi‐Tab ar, 2019). Yet, the ministry delegates its responsibilities to over 60 affiliated universities of medical sciences and health services across the country. There is at least one university of medical sciences and health services in each province whose president is the highest health authority in that province and reports to the Minister of Health and Medical Education. Moreover, the ministry and the affiliated universities of medical sciences and health services have the legal authority to oversee, licence and regulate the activities of the private health sector (Mehrdad, 2009).

Meanwhile, nursing forms the largest body of employees in the Iranian healthcare system, working across all segments of care. Nursing in Iran has witnessed profound advancements in the past few decades despite numerous issues and challenges that still exist (Ahmadi‐Chenari et al., 2020). Early nursing in Iran was influenced strongly by the British nursing tradition, characterized by an apprenticeship style of nurse education. However, this influence has been replaced largely by transferring all registered nursing education into the university‐based higher education sector. This change has led to a remarkable increase in scientifically educated nurses, quality and quantity of nursing researches, and qualified nursing faculties. Thereby, it has enhanced the professional status of nurses in Iran (Nasrabadi et al., 2004). Today, Registered Nurses must complete a four‐year bachelor's degree program as the minimum entry‐to‐practice requirement in the Iranian healthcare system. Other university‐based nursing programmes include two‐year master's degree programmes and four‐year doctoral degree programmes (Tabari‐Khomeiran & Deans, 2007). Currently, most of the nurses in Iran are females, hold a BSN degree and work in hospitals and healthcare centres owned and operated by the MOHME (Ahmadi‐Chenari et al., 2020). MOHME regulates nursing bachelor's degree programmes, licences schools and determines their curriculum. All schools are obliged to follow a centralized national curriculum, allowing for some flexibility in non‐core courses in this curriculum. This ministry also oversees master's and doctoral degree programmes which must be accredited to give professional degrees (Tabari‐Khomeiran & Deans, 2007).

On the other hand, Iranian nurses' 30‐year endeavour for recognition ultimately resulted in the establishment of the Iranian Nursing Organization (INO) that was approved by the parliament of Iran in 2002. Since then, this professional self‐regulatory organization has had the legal responsibility to develop standards for nursing practice, to improve the quality of patient care and to represent all nurses in the healthcare system of the country (Yadegary et al., 2017). The establishment of the nursing deputy (mainly responsible for making clinical nursing policies) and the board of nursing (mainly responsible for making educational nursing policies) in MOHME has also played a significant role in the development and empowerment of Iranian nurses over the last few years (Adib‐Hajbaghery & Salsali, 2005; Cheraghi et al., 2015). However, the public image of the nursing profession in Iran still does not appreciate nurses' work and education which contributes to poor morale and self‐image and frustration, hopelessness, and reduced job satisfaction and retention. It is increasingly recognized in Iran that the media contributes to the negative image that society holds about nursing. Iranian nurses are often not shown as helpful or competent in the media, and the media fails to present nurses as scientific and knowledgeable members of a medical team. In other words, despite many changes, nursing is still striving to be accepted and recognized as a profession by the general public in Iran (Ameri et al., 2018).

## 2 METHODS

### 2.1 Design

This descriptive‑correlational study was carried out as a nursing master thesis approved by the institutional review board and the research ethics committee of Tabriz University of Medical Sciences (TUOMS). Moreover, study objectives were explained to the hospital officials, and permissions were obtained from them before data collection.

### 2.2 Setting and participants

The study was conducted on a sample of nurses working at 12 university hospitals in Tabriz, Iran, from April–September 2018. The participants were chosen using a proportionate stratified random sampling technique. This was a combination of stratified sampling technique and proportional sampling technique followed by random sampling technique. First, the sample size of each university hospital was calculated proportionate to the population size of each university hospital. That is, the size of the sample drawn from each stratum is proportional to the relative size of that stratum. Following these calculations, the researchers performed simple random sampling in each of the 12 university hospitals to select participants using the lists of nurses drawn from each university hospital.

Study inclusion criteria were as follows: (a) having a minimum of a bachelor's degree in nursing, (b) having at least 6 months of clinical work experience and (c) currently working as a nurse in one of the study settings. Being unwilling to participate in the study and incomplete questionnaires were the exclusion criteria. None of the participants met the exclusion criteria, and no one was excluded from the study. Following sample size calculation from each university hospital, a pilot study was carried out using 30 randomly selected eligible nurses of 12 university hospitals in the city of Tabriz. This was to determine initial data for performing a proper sample size calculation and to test for the applicability and clarity of the data collection instrument before proceeding with the main study. The data from the pilot study were not included in this study because it represented an initial validation of our methods. In the G*Power 3.1.2 software, a two‐tailed test of the correlation coefficient in the bivariate normal model was used with the following input parameters (an alpha [*α*] error probability of .05, power of .8, correlation probability H_1_ of .174 and correlation probability H_0_ of zero) which yielded a sample size of 256 participants. Then, the calculated sample size was modified to 233 participants by finite formula (given the total population size of 2,625 nurses working at university hospitals of Tabriz) and assuming a non‐response rate of 10%. The final sample size was determined to be 250 participants.

### 2.3 Data collection and procedures

A three‐part questionnaire was used for data collection. The first part included Demographic Characteristics. The second part addressed the Porter Nursing Image Scale that was developed by Porter and Porter in 1991 to measure nurses' perception of the PI of nursing. The scale consists of 30 matched‐pair, bipolar adjectives, which are divided into three subgroups: interpersonal power (13 items), interpersonal relations (10 items) and intrapersonal ability (7 items). Interpersonal power items assess the professional aspect of nurses such as being a leader, independent and scientific. Interpersonal relation items measure caring attitudes and interactive aspects of nurses. Finally, the items in intrapersonal ability are concerned with the rationality of nurses. The scale was rated on a seven‐point semantic differential scale. The possible scores of each item ranged from 1–7, and the sum of each subject's scores in all items ranged from a possible 30–210. Lower scores indicate a negative perception of PI, and higher scores indicate a positive perception of PI (Porter & Porter, 1991). The third part was the Quality of Nursing Work Life (QNWL) Scale that was developed by Brooks in 2001 to determine nurses' working life quality. The scale consists of 42 items and it has four subscales: work/home life (Family), work design (Profession), work context (Organization) and work world (Community). Each item is scored on a 6‐point Likert scale ranging from “completely disagree (1 point)” and “completely agree (6 points).” Item 20 in the scale is reverse coded. The minimum total score is 42, and the maximum is 252. Higher total scores indicate better working life quality (Brooks, 2001).

The English version of the questionnaire was translated into a Persian version using forward and backward translation process (King et al., 2011), followed by content validity which were confirmed by a panel of experts consisting of 10 faculty members and 10 nurses of TUOMS. Some minor changes were applied according to expert recommendations. Besides, to evaluate the internal consistency and reliability of the final Persian versions of the instrument, Cronbach alpha was calculated as 0.84 for the Porter Nursing Image Scale and 0.89 for the Brooks QNWL Scale, based on the above‐mentioned pilot study.

During the course of the study, one of the researchers constantly visited different wards of the university hospitals and identified the eligible nurses. The objectives of the study were explained to all participants, and the questionnaires were collected at the completion of each shift.

### 2.4 Statistical analysis

Descriptive analysis included frequency and percentage for discrete data, mean and standard deviation (*SD*) for normally distributed continuous data, and median and interquartile range (IQR) for non‐normally distributed continuous data. The standard scores were also calculated for nurses’ perception of the PI of nursing and QNWL, so they can be compared. Pearson correlation test was also used for evaluating the relationship between nurses’ perception of the PI of nursing and QNWL. On the other hand, a multiple regression analysis was developed based on a two‐step hierarchical strategy. In the first step, a backward strategy using statistical differences analysis (including independent samples *t* test and one‐way analysis of variance (ANOVA) for parametric data and Mann–Whitney *U* test and Kruskal–Wallis *H* test for non‐parametric data) was applied across participant characteristics to determine significant baseline predictors of QNWL at *p*‐values of ≤.25. In the second step, all the candidate predictors were entered into the model. The final model consisted of significant baseline predictors and the scores of nurses’ perception of the PI of nursing. The regression assumptions of residual normality, homogeneity of residual variances, residual independence and co‐linearity were assessed and confirmed using normal probability plot, residual versus predicted values plot, Durbin‐Watson Statistics (values between 1.5–2.5 as the acceptable range) and Variance Inflation Factor (VIF<5 as the acceptable values). The level of significance was set at.05. Data were evaluated by the Statistical Package for the Social Sciences (SPSS) version 25 software (SPSS Inc).

### 2.5 Ethical considerations

The study was approved by the Institutional Review Board and the regional Research Ethics Committee of TUOMS (ethics code: IR.TBZMED.REC.1397.109). Moreover, permissions were obtained from the officials of the university hospitals. Each participant signed a consent form before the questionnaires were handed out. The questionnaires were completed in an anonymous manner, and respondents were assured of the confidentiality of their responses.

## 3 RESULTS

All distributed questionnaires were completed and returned (response rate = 100%). The mean age of participants was 33.4 ± 8.1 years, ranging from 22–56 years, and the average work experience was 9.3 ± 7.3 years, ranging from 1–30 years. Females made up 85.6%, 74.4% were married, 85% had a bachelor's degree, 84.4% were locals, and 74% worked rotating shifts. Moreover, 52.8% reported that they had an income lower than their expenses. Other demographics of the participants are listed in Table 1 which also presents the variables significantly associated with both the nurses' perception of the PI of nursing (*p *≤ .05) and their QNWL (*p *≤ .05).

**TABLE 1:** Participant's characteristics

Variable	No. (%)	Mean of PI scores ± *SD*	Test statistics	*p*‐value	Mean of QNWL scores ± *SD*	Test statistics	*p*‐value
Gender (*n* = 250)							
Female	214 (85.6)	120.95 ± 42.47	*U* = 2,875 *Z* = −2.434	.015^a^	143.87 ± 25.11	*t* = −1.928 *df *= 41.53	.061^b^
Male	36 (14.4)	144.28 ± 29.82			155.39 ± 34.33		
Age, years (*n* = 250)							
≤30	106 (42.4)	124.36 ± 39.65	*H* = 0.995 *df *= 2	.608^c^	150.77 ± 29.53	*F* = 2.452 *df *= 249	.034^d^
31–40	97 (38.8)	121.15 ± 43.11			140.61 ± 25.13		
≥41	47 (18.8)	127.67 ± 42.19			143.85 ± 21.92		
Marital status (*n* = 250)							
Single	64 (25.6)	123.69 ± 45.57	*U* = 5,685 *Z* = −0.535	.593^a^	145.53 ± 26.05	*t* = 0.001 *df *= 248	.999^b^
Married	186 (74.4)	123.75 ± 39.99			145.52 ± 27.20		
Education (*n* = 250)							
Bachelor	215 (86)	124.47 ± 41.06	*U* = 3,504.5 *Z* = −0.650	.515^a^	146.11 ± 27.59	*t* = 0.844 *df *= 248	.399^b^
Master	35 (14)	119.17 ± 43.75			141.97 ± 21.87		
Type of university attended (*n* = 250)							
State	130 (52)	121.31 ± 41.44	*U* = 7,300 *Z* = −0.875	.381^a^	146.51 ± 24.51	*t* = 0.595 *df *= 232.87	.552^b^
Private	120 (48)	126.36 ± 41.37			144.47 ± 29.27		
Residency (*n* = 250)							
local	211 (84.4)	122.10 ± 42.04	*U* = 3,541.5 *Z* = −1.381	.167^a^	144.42 ± 26.50	*t* = −1.518 *df *= 248	.130^b^
Non‐local	39 (15.6)	132.54 ± 37.04			151.51 ± 28.33		
Position (*n* = 250)							
Nurse	237 (94.8)	122.81 ± 41.59	*U* = 1,186.5 *Z* = −1.395	.163^a^	144.81 ± 27.33	*t* = −4.413 *df *= 25.54	<.001^b^
Head‐nurse	13 (5.2)	140.54 ± 35.08			158.69 ± 9.37		
Work shifts (*n* = 250)							
Fixed	43 (17.2)	128.28 ± 46.11	*U* = 3,959 *Z* = −1.139	.255^a^	149.93 ± 24.07	*t* = 1.182 *df *= 248	.238^b^
Rotational	207 (82.8)	122.79 ± 40.41			144.61 ± 27.37		
Work experience, years (*n* = 250)							
≤5	102 (40.8)	129.19 ± 39.06	*H* = 9.059 *df *= 3	.029^c^	147.31 ± 25.04	*F* = 1.190 *df *= 249	.314^d^
6–15	100 (40)	115 ± 43.47			145.68 ± 31.27		
16–25	40 (16)	127.52 ± 39.34			138.97 ± 20.36		
≥26	8 (3.2)	144.25 ± 38.98			153.62 ± 10.76		
Financial status (*n* = 250)							
Income=Expenses	89 (35.6)	123.53 ± 41.46	*H* = 1.485 *df *= 2	.476^c^	150.83 ± 26.39 ^a^	*F* = 15.777 *df *= 249	<.001^d^
Income˃ Expenses	29 (11.6)	114.83 ± 42.93			164.17 ± 31.85 ^b^		
Income<Expenses	132 (52.8)	125.83 ± 41.08			137.86 ± 23.05 ^c^		
Level of family support (*n* = 250)							
Very bad	27 (10.8)	122.11 ± 36.11	*H* = 6.763 *df *= 4	.149^c^	135.37 ± 27.61	*F* = 5.012 *df *= 249	.001^d^
Bad	16 (6.4)	137.37 ± 58.33			125.00 ± 22.16		
Moderate	98 (39.2)	117.39 ± 38.05			144.97 ± 27.01		
Good	69 (27.6)	124.36 ± 40.59			152.16 ± 26.93		
Very good	40 (16)	133.80 ± 44.79			150.52 ± 21.99		
Spouse's Education (*n* = 186)							
Diploma	17 (9.1)	102.23 ± 52.59	*H* = 9.681 *df *= 3	.021^c^	122.53 ± 16.45	*F* = 3.903 *df *= 185	.005^d^
Associate	17 (9.1)	150.53 ± 32.65			154.18 ± 13.95		
Bachelor	109 (58.6)	121.00 ± 39.18			146.92 ± 26.88		
Master	32 (17.2)	121.34 ± 27.83			146.41 ± 19.19		
Doctorate	11 (5.9)	149.82 ± 40.29			151.36 ± 35.67		
Spouse's job (*n* = 186)							
Employee	149 (80.1)	120.51 ± 37.32	*H* = 15.044 *df *= 5	.010^c^	146.44 ± 27.91	*F *= 0.818 *df *= 185	.138^d^
Retired	10 (5.3)	148.70 ± 51.94			153.60 ± 13.47		
Unemployed	3 (1.6)	83.33 ± 80.25			135.67 ± 27.13		
Worker	4 (2.2)	130.00 ± 32.33			127.50 ± 22.52		
Housewife	8 (4.3)	162.87 ± 28.34			138.62 ± 30.32		
Self‐employed	12 (6.5)	125.08 ± 41.30			140.58 ± 26.09		

^a^ Mann–Whitney *U* test.

^b^ Independent samples *t* test.

^c^ Kruskal–Wallis *H* test.

^d^ One‐way ANOVA.

Furthermore, the result of independent samples *t* test, one‐way ANOVA, Mann–Whitney *U* test and Kruskal–Wallis H test indicated that the following variables: the participants’ gender, age, position, work shifts, residency, financial status, level of family support, spouse's education and spouse's job were the potential predictors of QNWL (*p* ≤ .25), thus included in the multiple regression analysis.

As shown in Table 2, the total mean score of the nurses' perception of the PI of nursing was 123.7 ± 41.4 while the total mean score of the QNWL was 145.5 ± 26.9. Among the subscales of Porter Nursing Image Scale, “intrapersonal ability” had the highest mean standard score (53.6 out of 100), followed by “interpersonal relations” and “interpersonal power.” On the other hand, “work design (profession)” had the highest mean standard score (58.2 out of 100) among the subscales of Brooks QNWL Scale while “work world (community)” had the lowest mean standard score (41.6 out of 100).

**TABLE 2:** Mean scores of nurses’ perception of the PI of nursing and QNWL

Variables	Items	Min‐Max	Mean ± *SD*	95% CI	Mean of standard score (out of 100)
Nurses' perception of the PI of nursing					
Interpersonal power	13	13–91	52.64 ± 18.03	50.4–54.9	50.81
Interpersonal relations	10	10–70	41.59 ± 16.84	39.5–43.7	52.65
Intrapersonal ability	7	7–49	29.51 ± 11.54	28.1–30.9	53.59
Total	30	30–210	123.73 ± 41.40	118.6–128.9	52.07
QNWL					
Work/home life (Family)	7	11–37	22.32 ± 5.30	21.7–23	43.78
Work design (Profession)	10	28–55	39.13 ± 5.36	38.5–39.8	58.26
Work context (Organization)	20	34–110	68.66 ± 16.19	66.7–70.7	48.66
Work world (Community)	5	6–27	15.42 ± 4.05	14.9–15.9	41.68
Total	42	91–227	145.53 ± 26.86	142.2–148.9	49.29

Abbreviations: CI, confidence interval; Max, maximum; Min, minimum; *SD*, standard deviation.

All pairwise Pearson's correlation coefficients were calculated to evaluate the possible relationship between the subscales of both Porter Nursing Image Scale and Brooks QNWL Scale, as presented in Table 3. Accordingly, there was a significant positive correlation between nurses’ perception of the PI of nursing and QNWL (*r* = 0.158, *p *= .012).

**TABLE 3:** The relationship between nurses' perception of the PI of nursing and QNWL

Variable	QNWL				
	Work/ home life	Work design	Work context	Work world	Total
Nurses’ perception of the PI of nursing					
Interpersonal power	*r* = .037	*r* = .029	*r* = .125	*r* = .127	*r* = .096
	*p *= .557	*p *= .646	*p *= .048	*p *= .044	*p *= .129
Interpersonal relations	*r* = .038	*r* = .003	*r* = .12	*r* = .072	*r* = .09
	*p *= .551	*p *= .965	*p *= .058	*p *= .253	*p *= .154
Intrapersonal ability	*r* = .146	*r* = .072	*r* = .233	*r* = .222	*r* = .214
	*p *= .021	*p *= .257	*p *< .001	*p *< .001	*p *= .001
Total	*r* = .089	*r* = .022	*r* = .185	*r* = .162	*r* = .158
	*p *= .159	*p *= .728	*p *= .003	*p *= .01	*p *= .012

Finally, using the enter method, a multiple linear regression was calculated to predict QNWL based on nurses’ perception of the PI of nursing and all the candidate predictors with *p*‐values of ≤ 0.25 (including gender, age, position, work shifts, residency, financial status, level of family support, spouse's education and spouse's job). A statistically significant regression equation was found (*F*_(10,175)_ = 3.017, *p *= .001), with an *R* ^2^ of 15.2%. After adjusting other covariates, participant`s QNWL increased 0.058 for each score of nurses' perceptions of the PI of nursing (*β* = .086, *p *= .240).

## 4 DISCUSSION

Our study showed a positive relationship between the nurses' perception of the PI of nursing and the QNWL which implies that nurses' negative perception of the PI of their profession is associated with their low quality of work life. Nurses' perception of the PI of nursing along with other significant predictors including gender, age, position, work shifts, residency, financial status, level of family support, spouse's education and spouse's job significantly explained 14.7% of the predictability of QNWL. In addition, nurses' perception of the PI of nursing itself is able to explain 2.5% of QNWL variance. This finding is consistent with an interpretive impression of a previous study by Kalisch et al., revealing that a negative PI has an adverse impact on the quality and quantity of the work performed (Kalisch et al., 2007). Similarly, an Australian study carried out by Takase et al., (2006) investigated how nurses’ interpretations of the PI of nursing affect work behaviour and showed that perceived PI of nursing was strongly related to nurses' job performance and turnover intentions (Takase et al., 2006). Kemmer and Silva (2007) also concluded that a negative image diminishes nurses' ability to change the directions of health care (Kemmer & Paes‐da‐Silva, 2007).

Furthermore, this study showed that Iranian nurses' own perception of the PI of their profession was at a moderate range. However, the previous studies revealed a negative image of nursing in Iran and believed that the nursing profession is not well perceived by the public (Nasrabadi et al., 2003; Vaismoradi et al., 2011; Varaei et al., 2012). Varaei et al. (2012) described the domains of “characteristics required for entry to work,” “social role characteristics of nursing” and “prestige, economic, social status and self‐image” as the determinants of this negative PI in Iranian nurses (Varaei et al., 2012). A recent study conducted by Ameri et al. (2018) also uncovered the key role of the Islamic Republic of Iran Broadcasting in distorting the PI of nursing, as it left the viewers with a false negative and inaccurate outlook and mindset of the nursing profession (Ameri et al., 2018). Likewise, in Iranian television and media, nurses are hardly seen as a professional consultant or expert, but mostly as a feminine and physician‐subordinate career (Valizadeh et al., 2016a).

In Iran, there is a substantial gap between the image of the nursing profession as perceived by nurses and that of the public (Azadi et al., 2013). The Iranian public has a fuzzy image of nurses which derives from their lack of recognition and knowledge about nursing roles, responsibilities and competencies. This public's lack of awareness of the nature of current nursing work may partly be a result of the nurses' expansion into other healthcare fields, which has blurred professional boundaries between nursing and other healthcare professionals (Doiron et al., 2008; Lewis & Urmston, 2000). Also, there is still some adherence to the old traditional image of nursing in Iranian society considering nurses as an angel, handmaiden and/or battleaxe. Besides, part of the poor image is due to the semantic ambiguity of the term “Parastar,” the Persian form of the term “nurse” and the improper and unprofessional use of this term in Iran, since Iranians define “Parastar” as someone with limited or no education who provides any type of care, such as a ward orderly, hospital attendant, nursing assistant or someone caring for children or the elderly at home (Azadi et al., 2013).

On the other hand, the nurses' own perception of the PI of nursing is mostly positive. It can be inferred that this positive perception has partly resulted from recent advancement in the field of nursing education, practice and policy in the Iranian healthcare system. In particular, the establishment of the INO as a legal nursing organization, constitution of the Deputy of Nursing in Iran Ministry of Health and Medical Education, evolutions in nursing postgraduate education and social elite recognition of postgraduates, improvements in professional mobility of nursing, the presence of Iranian nurses in the legislative and executive body of government, and involvement of Iranian nurse leaders in policymaking for nursing and their selfless efforts (Heshmati‐Nabavi et al., 2014; Nejatian & Joulaei, 2018).

Unlike the previous studies in Iran, our study indicated a more positive PI in male nurses than female nurses which may be explained by better job opportunities and security for male nurses as compared to the other professionals at the current financial and economic crisis in Iran. Valizadeh et al. (2016) also stated that the Iranian patriarchal healthcare system helps male nurses move up the ladder through the support they receive from physicians, and offer them unique career insights with higher pay (Valizadeh et al., 2016a). Moreover, an extensive review of the literature shows that the actual PI of nursing is globally diverse and incongruous, depending on the country and the culture, nurses live and work in. However, this image is mainly positive in developed countries with advanced health care and nursing systems and partly negative in low‐ and middle‐income countries with less advanced healthcare and nursing systems (Kalisch et al., 2007, Kemmer & Paes‐da‐Silva, 2007, Dahlborg‐Lyckhage & Pilhammar‐Anderson, 2009, Ben‐Natan & Becker, 2010, de‐Araujo‐Sartorio & Pavone‐Zoboli, 2010, Katz, 2007, Donelan et al., 2008, Huffstutler et al., 1998, Karanikola et al., 2011, Ozakgul et al., 2017).

The finding that QNWL is not at a favourable level in Iran is consistent with existing literature (Moradi et al., 2014; Nayeri et al., 2011; Mohamadi et al., 2014; Fallahee‐Khoshknab et al., 2007; Habibzadeh et al., 2012; Dargahi et al., 2007). The results from another Iranian study reflected a positive significant relationship between the QNWL and the nurses’ productivity (Saber et al., 2013). Two recent studies also concluded that the QNWL can well predict the nurses’ burnout (Farsi et al., 2010) and tendency towards turnover (Almalki et al., 2012), that is, the higher the QNWL, the lower the burnout, the lower the tendency towards turnover. In studies carried out in the United States and Canada, it was found that income, autonomy and professional independence were among the important factors improving the quality of nursing work life. However, ambiguous organizational goals and policies and job stress significantly contributed to the nurses’ dissatisfaction with their quality of working life (Nowrouzi et al., 2016; Best & Thurston, 2006). It is noteworthy to mention that the understaffing; heavy workloads; unconventional work hours; lack of support from managers and supervisors; inequality and discrimination in practice and workplace; conflict with colleagues, managers, and doctors; lack of opportunities for advancement; lack of appreciation from superiors; and lack of appreciation from patients and their relatives are some of the major hurdles in Iranian nurses’ context of practice which explain their unfavourable quality of working life (Valizadeh et al., 2016a).

### 4.1 Limitations

Despite the strengths of this study, it has certain limitations. First, we only studied a sample of nurses working at university hospitals of a metropolitan city in the northeast of Iran and a broader range of nurses in other regions may produce different results. Therefore, a cautious interpretation and application of the findings are necessary and future studies with broader samples should be conducted in different regions of Iran and diverse populations in different cultures of the world. Another potential limitation is the self‐report nature of our questionnaire meaning that, in some cases, the participants might not have disclosed their real viewpoints and described their perception of the PI of nursing and/or their quality of working life to be better or worse than it really was. To combat this, we explained the objectives of the study and debriefed the measures taken to the study participants if required. Finally, the scales used in the questionnaire were developed in 1991–2001, and therefore relate to a time when nursing globally and culturally was very different. It would be useful if the questionnaire was updated for cultural relevance and incorporate more contemporary elements. Variations in the motivational characteristics, lived experiences, mental condition and cultural features of the participants were also of the factors influencing how they define the PI of nursing and quality of working life and might impact the results of the present study that, of course, were beyond the control of the researcher. Despite these limitations, however, we believe that the findings can be reasonably interpreted in terms of cultural differences between the countries concerned and provide an adequate snapshot of the QNWL for the study participants.

## 5 CONCLUSIONS AND IMPLICATIONS

This study overall revealed a positive relationship between the nurses' perception of the PI of nursing and their quality of work life. It implies that improving the PI of nursing leads to a corresponding increase in the quality of nursing work life. In this connection, it is a necessary requirement to sensitize the authorities about optimization of the image of the nursing profession in the public's view along with considering other significant predictors including gender, age, position, work shifts, residency, financial status, level of family support, spouse's education and spouse's job. Since the first step to creating a positive PI is to connect with your target society and improvements in the PI of nursing are contingent upon the promotion of community awareness of nurses' real roles, responsibilities and competencies in healthcare systems, it is highly recommended for the healthcare managers to continuously work on the establishment of the PI of nursing by approaching both the public and nurses. They should make the best and most use of mass media and public awareness campaigns to convey a stereotype‐free positive image of the nursing profession and to enhance the potency of nursing, considering that this image is vital to the profession, necessary in today's changing workplace and an important concept for future nurses to understand. Moreover, reflective practice, professional socialization and nurse empowerment are other ways to improve the nurses' both the psychological (nurses' perception of the PI of their profession) and functional (quality of nursing work life) states. Also, more effective steps should be taken to provide professional orientation for nurses and nursing students and motivate them to improve the PI of nursing through better performance.

## CONFLICT OF INTEREST

The authors declare no conflict of interest in this study.

## AUTHORS CONTRIBUTION

FR, AS, GM: original concept and study design; GM: data collection; AS, GM, NG: data analysis and interpretation; AS, GM, TW, HF: manuscript preparation and final critique; HH, FR, HF: study supervision. All authors have agreed on the final version and meet at least one of the following criteria (recommended by the ICMJE [https://www.icmje.org/recommendations/]): substantial contributions to conception and design, acquisition of data or analysis and interpretation of data; drafting the article or revising it critically for important intellectual content.

## ETHICAL STATEMENT

None to be declared.

## Data Availability

The data that support the findings of this study are available on request from the corresponding author. The data are not publicly available due to privacy or ethical restrictions.
